# Angina in Women without Obstructive Coronary Artery Disease

**DOI:** 10.2174/157340310790231608

**Published:** 2010-02

**Authors:** Kamakki Banks, Monica Lo, Amit Khera

**Affiliations:** 1 From the Donald W. Reynolds Cardiovascular Clinical Research Center, the University of Texas Southwestern Medical Center, Dallas TX; 2 From the Division of Cardiology, the University of Texas Southwestern Medical Center, Dallas TX; 3 From the Department of Internal Medicine, the University of Texas Southwestern Medical Center, Dallas TX

**Keywords:** Syndrome X, angina, women.

## Abstract

Angina in the absence of obstructive coronary artery disease, sometimes referred to as cardiac syndrome X (CSX), is a debilitating condition that disproportionately affects women. More than 50% of women evaluated for angina have non-obstructive disease by cardiac catheterization, although the total numbers of women affected by CSX are unknown. Varying clinical definitions and the lack of large scale epidemiologic studies focusing on this illness have resulted in limited knowledge about its risk factors, although there appears to be an association with black race, estrogen deficiency, and insulin resistance. Contrary to prior beliefs about the benign nature of this entity, these women suffer considerable morbidity with costly economic implications that approach the lifetime costs of healthcare utilization for those with obstructive coronary disease. Two prevailing hypotheses have emerged to explain CSX: the ischemic hypothesis detailing abnormal coronary microvascular function and the non-ischemic hypothesis describing altered pain perception and myocardial hypersensitivity. Treatment strategies have focused on both of these pathways with the main goal of improving symptoms. Beta blockers provide the most convincing evidence for benefit, with other antianginals having secondary roles. Other promising pharmacologic therapies include xanthine derivatives, estrogen replacement therapy, ACE inhibitors, and statin medications, among other emerging treatment options. Neurostimulation and lifestyle factors including exercise can also be beneficial in reducing symptoms. However, managing patients with CSX can be frustrating for both patients and physicians, as there is a lack of data regarding an optimal treatment algorithm including few large-scale randomized controlled trials to clarify effective therapies.

## INTRODUCTION

A staggering number of women undergo coronary angiography each year due to anginal symptoms, only to discover “normal” findings. It is now appreciated that these women face significantly greater morbidity than once believed, with an uncertain treatment course and high rates of medical utilization. The pathophysiology of chest pain in women without obstructive coronary disease represents a heterogeneous mix: some have cardiac chest pain that is non-ischemic, others have cardiac chest pain due to microvascular ischemia or abnormal pain sensation, and still others have chest pain of non-cardiac origin. All of these women suffer from symptoms of chest pain; however, the prognosis and therapeutic options differ widely [1]. The following is a concise review of the epidemiology, prognosis, disease mechanisms, and treatment options for chest pain in women without obstructive coronary artery disease (CAD).

## DEFINITIONS

In 1973, Kemp introduced the term cardiac syndrome X (CSX) to describe patients with exercise-induced angina and normal coronary angiograms [2]. However, the use of this term has not always been limited to this specific meaning. The classic definition involves effort induced angina-like chest pain, ST segment depressions on stress testing, and normal epicardial coronary arteries. A broader definition found in the literature simply includes angina-like chest pain with normal epicardial arteries. Others have advocated a more stringent definition of effort induced angina attributed to coronary microvascular dysfunction [3]. Patients with other cardiac pathology, such as cardiomyopathy, left ventricular hypertrophy and valvular heart disease, are often excluded from these definitions [4]. The varying definitions of this entity contribute to the conflicting reports in the literature regarding its frequency, risk factors, and treatment. For the purposes of this review, a distinction will be made between the broader definition of angina without obstructive coronary artery disease and CSX (angina, ischemic changes on stress testing, and normal coronary angiograms) when possible.

## EPIDEMIOLOGY

### Prevalence

The American Heart Association (AHA) has estimated that over 9 million people in the United States suffer from angina pectoris, which significantly impacts quality of life, ability to work, and costs to society [5]. Among patients who undergo coronary artery angiography for the evaluation of angina, a significant proportion of them, mainly women, are found to have normal-appearing epicardial coronary arteries. The Coronary Artery Surgery Study (CASS) registry was one of the earliest large registry databases to describe the frequency of these findings in approximately 25,000 patients undergoing coronary angiography for angina, of which 39% of women compared with 11% of men had normal coronary arteries [6]. More recently, data from 375,886 patients referred for angiography due to stable angina in the American College of Cardiology-National Cardiovascular Data Registry (NCDR) showed that the prevalence of non-obstructive disease was significantly higher in women (51%) than in men (32%) [7]. This high prevalence in women was again confirmed by the Women’s Ischemic Syndrome Evaluation (WISE) study, where 62% of women referred for angiography had non-obstructive CAD [8].

Similar to stable angina, women presenting with acute coronary syndromes are more likely to have non-obstructive CAD compared with men, with prevalence rates of approximately 20% and 10%, respectively [7]. Angina itself also appears to be more prevalent in women than men, based upon a recent international comparison of 31 countries. Using the Rose Angina Questionnaire, the researchers uncovered a fairly consistent higher female prevalence of angina across countries with pooled estimates of 6.7% in women vs. 5.6% in men [9]. While all of these studies shed light on the frequency of angina and normal angiograms in women, the prevalence of CSX is not well defined and can only be extrapolated from these findings.

### Associated Characteristics

Demographic and clinical factors associated with CSX have largely been derived from smaller mechanistic and observational studies. Reports suggest that women with CSX are older and more frequently postmenopausal [10], but other larger studies, such as the WISE cohort, describe these women to be younger and more commonly premenopausal than those with obstructive CAD [8]. Clearly, methodological differences, such as selection bias and reference populations, impact these discrepant findings. Currently, there are no large scale epidemiologic studies evaluating risk factors for classically defined CSX. However, a few proposed characteristics associated with women and CSX are worthy of mentioning.

#### Black race

Angina with normal coronary arteries is a more common finding among blacks compared with whites. In the NCDR registry, Shaw *et al*. reported that the highest rates of non-obstructive disease in those undergoing coronary angiography were found in black women compared with other ethnicities (59% black, 53% Hispanic, 50% white, 47% Asian, and 45% Native American) [7] (Fig. **1**). Using the CASS registry, Maynard *et al*. found that 67% of black women, compared to 54% of white women, had non-obstructive coronary artery disease, with similar associations found in men [11]. Matthew *et al*. evaluated 654 black patients referred for coronary angiography for suspected CAD and found that 47% of the population had either completely normal angiograms or non-obstructive lesions, a higher prevalence than in their previously studied white cohort [12]. Blacks in this population tended to be younger and had fewer traditional cardiovascular risk factors compared to others with obstructive CAD.

While not all studies have found similar ethnic differences [13], most studies support higher rates of angina and non-obstructive coronary disease in blacks compared to whites. Subtle differences in results between the studies may be due to differing definitions of non-obstructive disease and referral bias inherent in the study designs. Some hypothesize that the higher incidence of left ventricular hypertrophy and obesity in blacks may lead to decreased coronary vascular reserve and possibly angina [12].

#### Estrogen deficiency and hysterectomy

Both estrogen deficiency and hysterectomy have been associated with CSX. Rosano *et al*. studied a cohort of 107 women with anginal symptoms, normal coronary angiograms and positive exercise stress tests [10]. In 95 of the 107 women with CSX, chest pain symptoms began during the perimenpausal (n=32) or post menopausal (n=63) period. Symptoms typical of the perimenopausal period, such as hot flashes, migraines, and sleep disturbances were associated with chest pain symptoms in this population further supporting a connection between estrogen deficiency and CSX. Additionally, women with CSX were four times more likely to have had a prior hysterectomy than age-matched controls, the majority of which only had symptoms after the procedure.

Sarell *et al*. followed anginal symptoms, 17β-estrodial (E2) levels, and fingertip hyperemic responses to brachial artery occlusion one month before and two months after hormone replacement therapy [14]. Women with CSX had a higher prevalence of hysterectomy compared to the control group and also demonstrated improved hyperemic response and anginal symptoms after estrogen therapy. Similarly, the WISE investigators observed hysterectomy as a more frequent condition in those with non-obstructive CAD than those with one-, two-, or three-vessel disease [8].

These studies support the premise that estrogen deficiency may be an important risk factor for CSX and offer therapeutic implications for estrogen replacement therapy. Although not always differentiated in these studies, hysterectomy even without oopherectomy appears to be a risk factor for CSX for unclear reasons. Some researchers have suggested that hysterectomy causes secondary ovarian failure leading to estrogen deficiency, providing the link between hysterectomy and CSX [10].

#### Insulin resistance

Several early studies reported greater insulin resistance in those with CSX compared with reference populations [15-17]. In these small studies, there was a greater rise in insulin levels in response to a glucose challenge in those with CSX than in controls, and of comparable magnitude to those with obstructive CAD [16, 17]. More recently, Jadhav *et al*. compared 52 women with CSX to 24 healthy age-matched controls and found higher insulin levels in those with CSX [18]. Patients with CSX also more commonly met diagnostic criteria for the metabolic syndrome (30% vs. 8%) and had a higher mean body mass index (28.6 vs. 25.1 kg/m^2^) [18]. Whether these latter two entities solely reflect insulin resistance or whether they are independently correlated with CSX is unclear.

## OUTCOMES

Until recently, the prognosis for angina in the absence of coronary artery disease was considered benign. This view was supported by small international observational and case-control studies beginning in the 1960s [19-24]. In general, angina in the presence of normal or near normal coronary arteries conferred no additional risk for myocardial infarction or cardiac death in these studies. However, they were limited by small sample size or relatively short follow-up period. Additionally, the heterogeneous grouping of non-obstructive disease with completely normal coronary arteries, makes the generalizability of these results difficult [2, 19-24].

A more recent report from a registry cohort of 32,000 patients undergoing coronary angiography in British Columbia, Canada, also confirmed the relatively good prognosis in terms of hard cardiovascular outcomes in such patients. Of all patients with normal coronary angiograms, death rate and stroke rate at one year were only 1% and 0.6% respectively. Of note, a portion of these patients underwent angiography due to acute coronary syndrome, which measurably increases subsequent vascular risk [25].

While CSX may not translate into increased cardiovascular mortality, recent reports from the WISE investigators have called attention to the considerable morbidity encountered by women with this condition [26, 27]. In an early substudy of 74 women with three-years of follow up, there were no deaths or myocardial infarctions in women with angina and non-obstructive CAD, but they still had considerable cardiovascular event rates (19%), consisting of hospitalizations for angina (16%) and repeat angiography (7%) [27]. Women with persistent chest pain were three times more likely to have cardiac events compared with those without such symptoms. In a more recent report, WISE participants with angina and normal coronary arteries (n=318) or nonobstructive CAD (n=222) were compared with 1000 age and race matched controls from a community based sample of women free of heart disease [26]. WISE women with normal coronary arteries had a more than three-fold increase in composite cardiovascular events (2.4% vs. 7.9%, adjusted p=0.002) over five years, including higher rates of stroke and heart failure hospitalizations. However, there was no statistical difference in rates of myocardial infarction (0.7% vs. 0.9%) or cardiac death (0.6% vs. 1.5%) despite numerical trends. Of note, WISE women with nonobstructive CAD had significantly higher all cause mortality rates than the control group (2.1 vs. 3.0%, p=0.04). These findings were echoed by the British Columbia registry where women with angina and normal coronary angiograms were four times more likely to be re-admitted for chest pain and acute coronary syndromes than similar men during early follow-up [25]. Importantly, while the presence and severity of atherosclerotic lesions among those with non-obstructive disease relate to outcomes and while these trials differed in criteria as to whether patients had normal angiograms or non-obstructive CAD, both demonstrated a high rate of recurrent cardiovascular events in these women [25-27].

The prognosis of women with chest pain without obstructive CAD or with CSX may be adversely impacted by the presence of concomitant coronary microvascular dysfunction [28-31]. Studies have suggested that CSX patients with this accompanying condition are more likely to develop atherosclerotic CAD in the future and have higher cardiovascular event rates [32, 33]. In fact, the WISE investigators stratified patients without obstructive CAD into those with and without myocardial ischemia on magnetic resonance spectroscopy. Those with evidence of myocardial ischemia, presumably from microvascular dysfunction, had significantly greater frequency of adverse cardiovascular events, even after accounting for accompanying risk factors [27].

### Economic Burden 

Current statistics suggest a substantial economic burden from angina in women without obstructive disease. In the U.S. more than four million women suffer from angina, accounting for a significant proportion of current cardiovascular healthcare costs. Over 500,000 women undergo coronary angiograms annually, of which >50% will reveal non-obstructive CAD, leading to an annual excess expenditure of $280 million [27, 34]. This figure does not include the cost of continued medical evaluation or care for such women with persistent symptoms and disability.

**Fig. (2). Estimated lifetime healthcare costs for cardiovascular disease in women from the WISE study.**
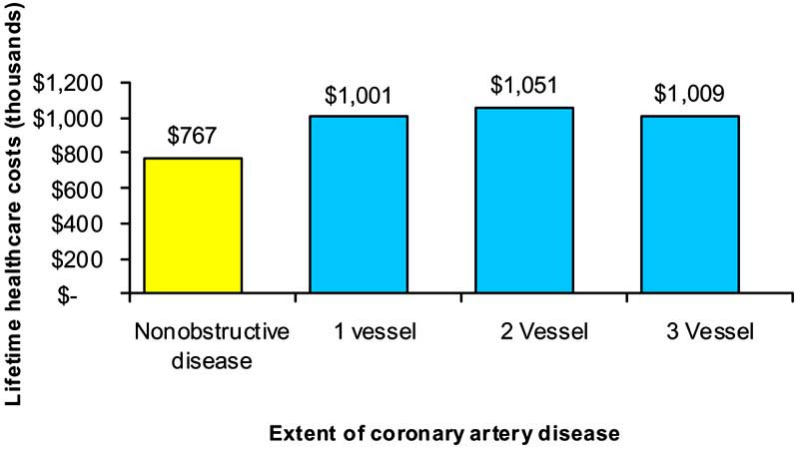
Adapted from Shaw *et al.* Circulation 2006 [8].

Recently, the WISE researchers performed a cost analysis for re-hospitalization, cardiac procedures, pharmaceuticals, and cardiac complications for 883 women referred to angiography for ischemic symptoms [8]. After one year, women with non-obstructive disease had a 1.8-fold higher rate of repeat angiography compared to those with single-vessel CAD. Additionally, 46% of women had persistent symptoms at five-year follow-up, and one of every five women was re-hospitalized for such symptoms. The estimated lifetime costs of healthcare utilization in women with chest pain and non-obstructive disease were $767,288 (Fig. **2**). Although this sum was lower than in women with single- and multi-vessel disease, it represents an underappreciated societal cost for the care of non-obstructive coronary disease with considerable economic implications (Fig. **2**).

## DISEASE MECHANISM 

### Ischemic Hypothesis

Two prevailing hypotheses have emerged to explain CSX and have been reviewed extensively elsewhere [3, 4, 35]: the ischemic hypothesis detailing abnormal coronary microvascular function and the non-ischemic hypothesis describing mainly altered pain perception and myocardial hypersensitivity. For a subset of women with ischemic changes on stress testing and other objective evidence of ischemia, microvascular dysfunction appears to be the prevailing pathophysiologic explanation for CSX. Both endothelium-dependent mechanisms as evaluated by coronary blood flow response to acetylcholine or pacing, and endothelium-independent pathways using coronary blood flow response to adenosine, contribute to this entity [35, 36]. Endothelial dysfunction leads to an imbalance between vasodilator substances, namely nitric oxide, and vasoconstrictor substances such as endothelin 1, as well as decreased release of anti-inflammatory and anti-thrombotic factors [37]. As such, not only is there impaired vasodilation to various stimuli, but several studies have demonstrated enhanced vasoconstriction in some patients with CSX [38-40]. Because microvascular dysfunction cannot be diagnosed by conventional coronary angiography, it is measured indirectly by invasive methods (thermodilution, coronary flow reserve) or by non-invasive methods assessing myocardial ischemia (radionuclide perfusion, PET, MRI scans) [4]. Indeed, recent studies using MRI scanning have demonstrated reduced subendocardial perfusion in subjects with CSX compared with controls [29]. The prevalence of microvascular dysfunction as assessed by ischemia on gated single-photon emission computed tomography or positron emission tomography is consistently 50%-60% in women with angina and normal or near-normal coronary arteries. Using MRI, approximately 25% of patients with angina and non-obstructive CAD have decreased coronary flow reserve. This prevalence may be underestimated due to the limited ability to achieve adequate levels of stress in the MRI magnet [29, 30].

Traditional cardiovascular risk factors, including hypertension, hypercholesterolemia, smoking, and diabetes, probably contribute to coronary microvascular dysfunction, particularly through impairment of endothelium-dependent vasodilatation [41]. Other abnormalities associated with microvascular ischemia include insulin resistance [15], estrogen deficiency in women [42], and low grade inflammation, as evidenced by increased level of C-reactive protein and the interleukin-1 receptor antagonist in patients with CSX [43].

### Non-Ischemic Hypothesis

The non-ischemic hypothesis describes altered pain perception as an etiology of CSX. Previous research has demonstrated that patients with chest pain and normal coronary arteries have increased pain sensitivity to peripheral stimuli including electrical and thermal skin stimulation [44, 45]. Recent advances in accurate neural and metabolic imaging techniques provide added insight into to this theory. There is evidence that habituation to repeated painful nociceptive stimuli is absent in patients with CSX [46]. In addition, since estrogens are known to have analgesic properties and act on the µ opioid system, it has been proposed that hormonal imbalances in postmenopausal women with CSX may influence pain perception and that the lack of estrogen may enhance the perception of chest pain [47]. Repeated episodes of myocardial ischemia and systemic inflammation may promote functional alterations in cardiac afferent nerve fiber endings, establishing a link between coronary microvascular abnormalities and increased pain sensitivity in CSX [46].

## DIAGNOSIS

Although CSX is often a diagnosis of exclusion, having a high clinical suspicion will help in the recognition of this syndrome. First, non-cardiac etiologies of chest pain, including musculoskeletal, psychiatric, gastrointestinal, and pulmonary disorders, must be excluded. Those with angina-like chest pain, and even some with atypical features, including more frequent or persistent pain [48] and inconsistent response to sublingual nitrates, should undergo stress testing. Down-sloping ST-segment depression remains a diagnostic criterion for classical CSX, although the broader definition of demonstrable myocardial ischemia on perfusion testing is also used clinically. While some have suggested that most stress testing characteristics in CSX are indistinguishable from changes seen in patients with coronary artery disease [4], certain characteristics may help identify patients with CSX such as ST-segment depression at a higher rate-pressure product among others [49].

After demonstrating evidence of myocardial ischemia, patients should undergo cardiac catheterization or other direct imaging of the coronary arteries to confirm the presence or absence of significant epicardial atherosclerotic stenosis. The use of controlled vasoconstrictor stimulation with ergonovine or acetylcholine has been proposed to rule out coronary artery spasm as a cause of chest discomfort [35]. However, the procedure is not without risks and is often not done. A case can be made that all such patients should undergo testing to detect coronary microvascular dysfunction for a more definitive, restrictive diagnosis [3]. Currently, such techniques are not widely used clinically and the optimal method for such testing has not been clearly defined. Due to the lack of a uniform diagnostic criterion and a reliable diagnostic test, pinpointing patients with CSX remains difficult.

## THERAPY

Managing patients with CSX can be frustrating for both patients and physicians, as there is a lack of data regarding an optimal treatment algorithm. Most studies are observational or only involve a small sample size, yielding conflicting results. The myriad of therapy options are not always efficacious for an individual patient and often require frequent re-assessment and individual tailoring. The main goals of treatment are to improve symptoms, mainly by using anti-anginal therapies, but also through pain modulation. Given the overall favorable prognosis for hard cardiovascular outcomes, it is unclear whether agents such as aspirin, statins, anti-hypertensive medications, etc. that modify cardiovascular outcomes have an additional role in this entity. Below is a detailed discussion of the therapeutic modalities now being utilized. Unless otherwise specified, the data presented involve both genders and subjects with a strict definition of CSX (angina, evidence of ischemia during stress testing, and normal coronaries on cardiac catheterization) (Table **1**).

### Pharmacotherapy

#### Beta Blockers

Multiple studies have demonstrated a central role for beta-blockers in the management of CSX [50-52]. Bugiardini *et al*. demonstrated in a small 16 subject, double-blinded, cross-over design that over a 7 day treatment period, propranolol significantly reduced the ischemic burden with improvement in ST-segment depression during continuous electrocardiographic monitoring, whereas verapamil did not [52]. Atenolol also decreased anginal symptoms, delayed or eliminated ischemic electrocardiographic changes on exercise treadmill test, and improved Doppler derived indices of left ventricular filling in a single-blinided, randomized, cross-over study by Fragasso *et al.* [51]. In another small double blind cross-over study involving 10 subjects, Lanza *et al*. showed that atenolol and amlodipine both improved quality of life compared with oral nitrates,after 4 weeks of administration. However, atenolol was shown to be more effective for symptomatic relief when compared with amlodipine [50]. More recently, Togni *et al*. evaluated nebivolol, a highly selective beta1-adrenergic receptor-blocker that increases endothelial nitric oxide availability, and its effect on coronary flow reserve. Intracoronary nebivolol improved coronary flow reserve in normal subjects with and without CAD on angiogram [53]. Although the response to beta-blockers is variable in improving chest pain (19-60%) [54], beta-blockers work by lowering adrenergic tone and reducing myocardial oxygen demand, as well as enhancing endothelium-dependent vasodilation. As such, beta blockers are considered first line therapy for CSX.

#### Calcium Channel Blockers

Clinical results from the use of calcium channel blockers are highly variable. While some studies suggest clinical benefit in CSX [55], others found no improvement in ischemic episodes [52]. Cannon *et al*. performed a 26-subject, randomized, double-blind, placebo-controlled study in which one-month therapy with the calcium channel blockers verapamil or nifedipine resulted in significant improvement in angina and exercise tolerance in those with reduced coronary vasodilator reserve [55]. As discussed above, Bugiardini’s study comparing propranolol and verapamil revealed that the beta-blocker had a more favorable effect than the calcium channel blocker [52]. There are few data to support the use of diltiazem in this condition [56], and despite favorable responses such as improvement in exercise-induced ST segment depressions with nifedipine, it has also been shown to worsen clinical symptoms [57]. Similarly, amlodipine has not proven to be effective in controlling chest pain in patients with CSX [50]. The efficacy of calcium channel blockers has yet to be established but can be used as combination therapy with beta-blockers.

#### Nitrates

The use of nitrates in CSX is controversial, as therapy with these agents have a high treatment failure rate. Observational studies by Kaski *et al*. have suggested the efficacy of sublingual nitrates for treating CSX, though this was effective in only 42% of the patients [58]. Others have reported greater benefit of nitrates in those with obstructive CAD and actually worsening of exercise tolerance with the use of nitrates for CSX [59]. In general sublingual nitrates may benefit symptomatic episodes, but long acting nitrates have proven disappointing as an initial treatment strategy and are best used as combination agents in patients with CSX [50].

#### Xanthine Derivatives

Some investigators have suggested a role for xanthine derivatives, such as oral aminophylline, theophylline and bamiphylline, which block the adenosine receptor and lead to a more favorable redistribution of coronary blood flow [35, 60]. In a placebo-controlled study, intravenous aminophylline improved exercise duration, decreased chest pain during exercise, and improved ST segment depression during exercise [60]. Acute administration of oral aminophylline has also been shown to improve the time to exercise induced angina, time to ST depression, and the magnitude of ST depression in patients with CSX [61], while there was no improvement with nitrates in the same popu-lation. This treatment may be particularly useful for patients with CSX as well as chronic obstructive airway disease or asthma.

#### Estrogen

As stated earlier, estrogens may have an effect on pain perception and can improve endothelium-dependent coronary vasodilation. Even though many patients with CSX are peri- or post-menopausal, studies regarding the efficacy of hormone replacement therapy using various preparations (transdermal estrogen, conjugated equine estrogens, or estrogen and progesterone combinations) provide conflicting results. In a cross-over trial of 25 post-menopausal women with CSX receiving a 17β-estradiol patch and placebo for eight weeks each, chest pain episodes decreased in frequency while receiving hormonal therapy [47]. However, there was no improvement in exercise duration. In a more recent double-blinded, randomized, placebo-controlled trial, use of 17β-estradiol, combined with a novel progestin (drospirenone) resulted in a mild to moderate reduction of anginal episodes, as well as improved myocardial perfusion reserve [62]. In summary, estrogen therapy may be considered for symptom management in postmenopausal women with CSX, balancing the slightly increased risk of CV events.

#### ACE Inhibitors

Given the reported role of the renin-angiotensin system in promoting microvascular dysfunction, angiotensin-converting enzyme (ACE) inhibitors have been proposed as potential therapies for CSX. In a small placebo-controlled trial, enalapril improved exercise treadmill performance in such patients [63]. More recently, similar benefits have also been shown with cilazapril, an agent not currently available in the United States [64]. The putative mechanisms for these benefits are enhanced nitric oxide (NO) production by increasing precursors such as L-arginine and decreasing nitric oxide inhibitors, namely asymmetric dimethylarginine (ADMA). In support of these beneficial effects on microvascular function, one study reported improved coronary flow reserve in patients taking enalapril [65]. Interestingly, studies of irbesartan, an angiotensin receptor blocker, have not been as favorable [66].

#### Statins

Outside of their lipid lowering properties, statins have been shown to decrease inflammation and improve endothelial function [28, 33]. As such, they are attractive agents for the management of CSX, which is commonly associated with elevated C-reactive protein levels. Small placebo controlled trials have demonstrated beneficial effects of various statins on exercise duration and time to ST segment changes during exercise treadmill testing [67, 68]. Importantly, these effects were not reserved to hypercholesterolemic patients [67]. In addition, brachial artery flow-mediated dilation (FMD), a measure of endothelium-dependent vasodilator function, improved by approximately 50% with statin use in some patients with CSX [69].

The combination of an ACE inhibitor and a statin may amplify the salutary effects on microvascular function by inhibiting oxidative stress, thereby improving endothelial function. One study randomized 45 subjects with CSX to the combination of ramipril and atorvastatin or to placebo for six months. At follow-up, only those that received ramipril and atorvastatin had increased FMD, as well as improved quality of life and exercise duration [68].

### Pain Modulation

Another way to improve symptoms experienced by patients with CSX is through pain modulation. Imipramine, typically used in chronic pain syndromes, blocks norepinephrine reuptake and enhances the inhibitory action of pain-modulating neurons. In a placebo-controlled, double-blinded study done by Cannon *et al*., 60 subjects were randomized to clonidine, imipramine, or placebo for three weeks [70]. Imipramine reduced chest pain by approximately 50%; however, subjects with esophageal dysmotility or psychiatric issues were not excluded. Additionally even at low doses, imipramine has significant anti-cholinergic side effects such as dizziness, nausea, and fatigue that contribute to a poor quality of life despite improvement in chest pain [71]. Spinal cord stimulation (SCS) via an implantable device can be an effective option for some patients. This technique delivers low voltage electrical impulses onto the dorsal column of the spinal cord, resulting in central and peripheral pain modulatory effects. It also alters cardiac autonomic function, thereby improving microvascular function and reducing ischemia. Most studies involving spinal cord stimulation involve small cohorts that are limited by a lack of control group, but suggest an improvement in anginal symptoms in the majority of patients which is durable for at least several months to years [72, 73].

A related procedure called transdermal electric nerve stimulation (TENS) has also demonstrated encouraging results. Studies showed that TENS increases coronary blood flow without changing the epicardial vessel diameter, thus exerting its effect on the microvasculature [74, 75]. In a cohort of 36 patients who had angina refractory to medication, neurostimulation regardless of technique (TENS or SCS) provided sustained pain relief [76]. Of note, some patients may not tolerate TENS well secondary to skin irritation.

### Novel Therapies

#### Asymmetric Dimethylarginine 

More recently, there has been interest in modulators of the vasodilator nitric oxide (NO) as contributors to endothelial dysfunction in CSX. ADMA, an inhibitor of NO synthase, is elevated in patients with CSX. In contrast, L-arginine, a precursor of NO, augments vascular dilation and the ratio of L-arginine to ADMA provides an index of systemic NO metabolism. In a study of 9 patients with CSX and 14 control subjects, the continuous infusion of L-arginine increased basal forearm blood flow and reversed the effects of ADMA [77]. In addition, Palloshi *et al*. provided 13 hypertensive patients with CSX oral L-arginine, which improved angina, resting systolic blood pressure, and quality of life [78].

#### Metformin

As many patients with CSX are insulin resistant [15], leading to endothelial dysfunction and microvascular ischemia, insulin sensitization via metformin may be beneficial. Indeed, a recent randomized, double-blind, placebo-controlled trial demonstrated that metformin may improve vascular function and decrease myocardial ischemia at 8-week follow up in 33 non-diabetic women with CSX [79].

#### Others Therapies

Other pharmacological therapies that have been considered but failed to demonstrate a benefit in CSX include α-antagonists (doxazosin or clonidine) [80] and the piperazine calcium channel blocker lidoflazine [81]. Novel drugs which may have activity include trimetazidine (partial fatty acid oxidation inhibitor), bosentan (ET-1 inhibitor), cariporide (Na-H+ exchanger), and fasudil (rho-kinase inhibitor). However, despite mechanistic plausibility, there are currently no data supporting the use of any of these agents for the treatment of CSX.

Nicorandil, an activator of vascular potassium channels that has coronary vasodilator properties, warrants further study in patients with CSX. Yamabe *et al*. demonstrated that nicorandil injection significantly improved anginal symptoms and ST segment depressions in those with small vessel disease by augmenting coronary blood flow [82]. In another small randomized, placebo-controlled, double-blind trial by Chen *et al*., oral nicorandil moderately ameliorated exercise-induced angina in those with microvascular ischemia [83]. Ranolazine, a novel anti-anginal agent shown to be efficacious for patients with CAD and refractory chest pain, is currently being studied in patients with CSX.

Basal superoxide production by circulating mononuclear cells contributes to intravascular oxidative stress and predicts future cardiovascular events in patients with CSX [84]. Allopurinol, a xanthine oxidase inhibitor, reduces vascular oxidative stress and vascular inflammation, thus theoretically improving endothelial function. It is currently being evaluated in the ongoing APEX study (Effects of Allopurinol on Coronary and Peripheral Endothelial Function in Patients with Cardiac Syndrome X) of patients with CSX.

Another therapy used for refractory angina that holds promise for CSX is extracorporeal enhanced counter pulsation (EECP). This treatment involves the sequential inflation and deflation of a series of cuffs on the lower extremities which is thought to improve endothelial function, amongst other beneficial effects, by mimicking an exercise training effect of shear forces on the vasculature. In fact, a recent small case series demonstrated a sustained improvement in anginal symptoms with EECP in this population [85].

### Lifestyle Modification

Lifestyle modification is an integral component of therapy for patients with CSX, despite limited studies to date evaluating its role. At minimum, lifestyle therapies will favorably impact the adverse cardiovascular risk factor profile in many patients with CSX, but may also have additional beneficial effects specific to this disease. Exercise training improves angina, exercise capacity, and mortality in patients with coronary disease and can similarly improve symptoms in patients with CSX, as shown in a small study by Eriksson *et al*. [86]. The increased coronary blood flow during exercise stimulates NO release from the vasculature and improves endothelial function [87]. Additionally, Asbury *et al*. explored cardiac rehabilitation as a treatment for CSX and found improved exercise tolerance, quality of life, chest pain symptoms, and psychological morbidity with an 8-week phase III cardiac rehabilitation program [88].

Given the association of obesity [89] and smoking [90] with endothelial dysfunction, weight loss and smoking cessation are encouraged in patients with CSX. Furthermore, low fat and Mediterranean style diets also have demonstrable effects on endothelial function [91]. Psychological intervention, such as cognitive behavioral therapy and group therapy, may be helpful for patients with resistant symptoms by teaching approaches to pain management [4].

## RECOMMENDATIONS

Awareness of CSX is imperative, as it is quite prevalent, accounting for up to 60% of the female population undergoing cardiac catheterization for angina. Once non-cardiac sources of chest pain are ruled-out and a positive stress test is found, the patient should undergo either non-invasive imaging (computed tomography or magnetic resonance imaging) or invasive imaging (cardiac catheterization) of his/her coronaries. If normal coronary arteries or non-obstructive disease are identified, the presumptive diagnosis of CSX can be made, although additional assessment of coronary microvascular function when feasible may provide a more definitive diagnosis and have prognostic implications. The initial management begins with lifestyle modification, as well as CAD risk factor reduction, including the empiric use of statins and ACE inhibitors in those with borderline or elevated risk factors. Pharmacotherapy focused on improving symptoms starts with beta-blockade, and the addition of nitrates and calcium channel blockers should be considered for second line therapy. Additional agents that can be considered include estrogen replacement therapy in postmenopausal women and metformin in those with impaired fasting glucose or impaired glucose tolerance. Refractory cases may benefit from referral to a structured cardiac rehabilitation program for supervised exercise training, or to a center with EECP capabilities. Imipramine may also be considered in such cases, as well as neurostimulation via TENS or implantation of spinal cord stimulators for those with persistent debilitating symptoms.

It has become quite clear that CSX is not a benign illness in terms of long term morbidity and large, randomized controlled trials to elucidate effective therapies for these patients are sorely lacking. Practitioners and researchers should adhere to and utilize a uniform definition of CSX in their clinical practice and studies, and clearly delineate when they are evaluating CSX versus the broader entity of chest pain with normal angiograms. Further studies are needed to better define risk factors associated with CSX to aid in the identification of affected individuals and to treat the root causes of the disease. Also, these characteristics may aid in the decision as to whether certain patients can undergo non-invasive angiography with CT scanning for evaluation of angina and positive stress tests given the large proportion of normal invasive tests in these women.

## Figures and Tables

**Fig. (1). Prevalence of significant coronary artery disease by gender and ethnicity in patients with stable angina symptoms undergoing angiography.**
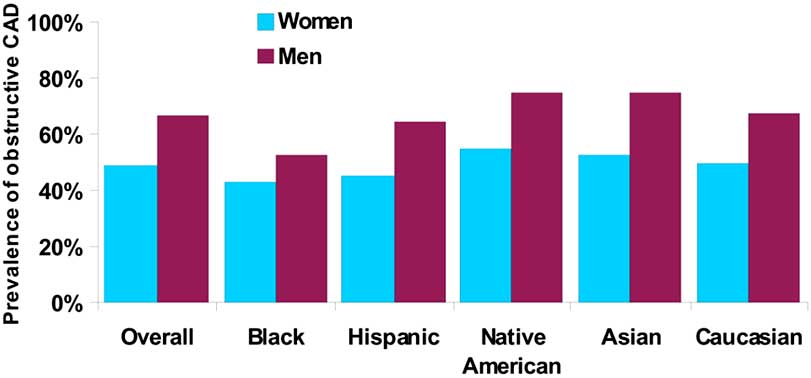
CAD = coronary artery disease Significant CAD defined as > 70% obstruction in at least one epicardial artery Adapted from Shaw *et al.* Circulation 2008 [7].

**Table 1.** Proposed Therapeutic Options Based on Efficacy

Therapy	Formulation	Efficacy
Nitrates		
	Oral	+/-
	Sublingual	+ (acute setting)
Beta-blocker		
	Propranolol	++
	Atenolol	++
Calcium-channel blockers		
	Verapamil	+/-
	Diltiazem	+/-
	Amlodipine	+/-
	Nisoldipine	+/-
	Nifedipine	-
Xanthine derivatives		
	Aminophylline (intravenous)	++
	Aminophylline (oral)	+
Hormone replacement		
	Estrogen	+
ACE inhibitors		
	Enalapril	+
	Cilazapril	+
	Ramipril	+ (when combined with statin)
ARB		
	Irbesartan	-
Statins		
	Simvastatin	+
	Atorvastatin	+ (when combined with ramipril)
Pain Modulation		
	Imipramine	+/- (relieves chest pain) (worsened overall quality of life)
	Neurostimulation (SCS/TENS)	++
Other pharmacological therapies		
	L-arginine (intravenous)	+
	L-arginine (oral)	++
	Metformin	+
	Doxazosin	-
	Clonidine	-
	Lidoflazine	-
	Nicorandil	++
	Ranolazine	?
	Allopurinol	?
Lifestyle/physiologic modification		
	Exercise	++
	Cardiac rehabilitation	++
	EECP	++

(-): No benefits shown

(+/-): Conflicting results

(+): Benefits in hemodynamic or stress testing profile

(++): Benefits in symptomatic relief

ACE indicates angiotensin converting enzyme inhibitors; ARB, angiotensin receptor blocker; SCS; TENS, transdermal electric nerve stimulation; EECP, extracorporeal enhanced counterpulsation;
